# β-carboline derivative Z86 attenuates colorectal cancer cell proliferation and migration by directly targeting PI3K

**DOI:** 10.1007/s13659-023-00422-y

**Published:** 2024-01-03

**Authors:** Shiyun Nie, Lizhong Chang, Ying Huang, Heyang Zhou, Qianqing Yang, Lingmei Kong, Yan Li

**Affiliations:** Key Laboratory of Medicinal Chemistry for Natural Resource, Yunnan Key Laboratory of Research and Development for Natural Products, School of Pharmacy, Ministry of Education, Yunnan University, Kunming, 650500 People’s Republic of China

**Keywords:** Colorectal cancer, PI3K, Z86, Proliferation, Cell cycle arrest

## Abstract

Phosphoinositide 3-kinase (PI3Ks) are lipid kinases widely involved in cell proliferation, metastasis and differentiation. Constitutive activation of the PI3K/Akt/mTOR signaling are well confirmed in colorectal cancers (CRCs). In this study, we identified isopropyl 9-ethyl-1-(naphthalen-1-yl)-9 H-pyrido[3,4-b] indole-3-carboxylate (Z86), as a novel PI3Kα inhibitor with the IC_50_ value of 4.28 µM. The binding of Z86 to PI3Kα was further confirmed with DARTS and CETSA assay. Immunofluorescence analysis and western blotting data demonstrated that Z86 effectively attenuated PI3K/AKT pathway. Z86 caused dramatic proliferation inhibition of CRCs through G0/G1 cycle arrest rather than apoptosis induction. Besides, the migration of CRCs was also relieved by Z86. The present study not only identified Z86 as a novel PI3Kα inhibitor with potent inhibitory efficiency on PI3K-mediated CRCs growth and migration, but also elucidated a reasonable molecular mechanism of Z86 in the Wnt signaling pathway inhibition.

**Graphical Abstract:**

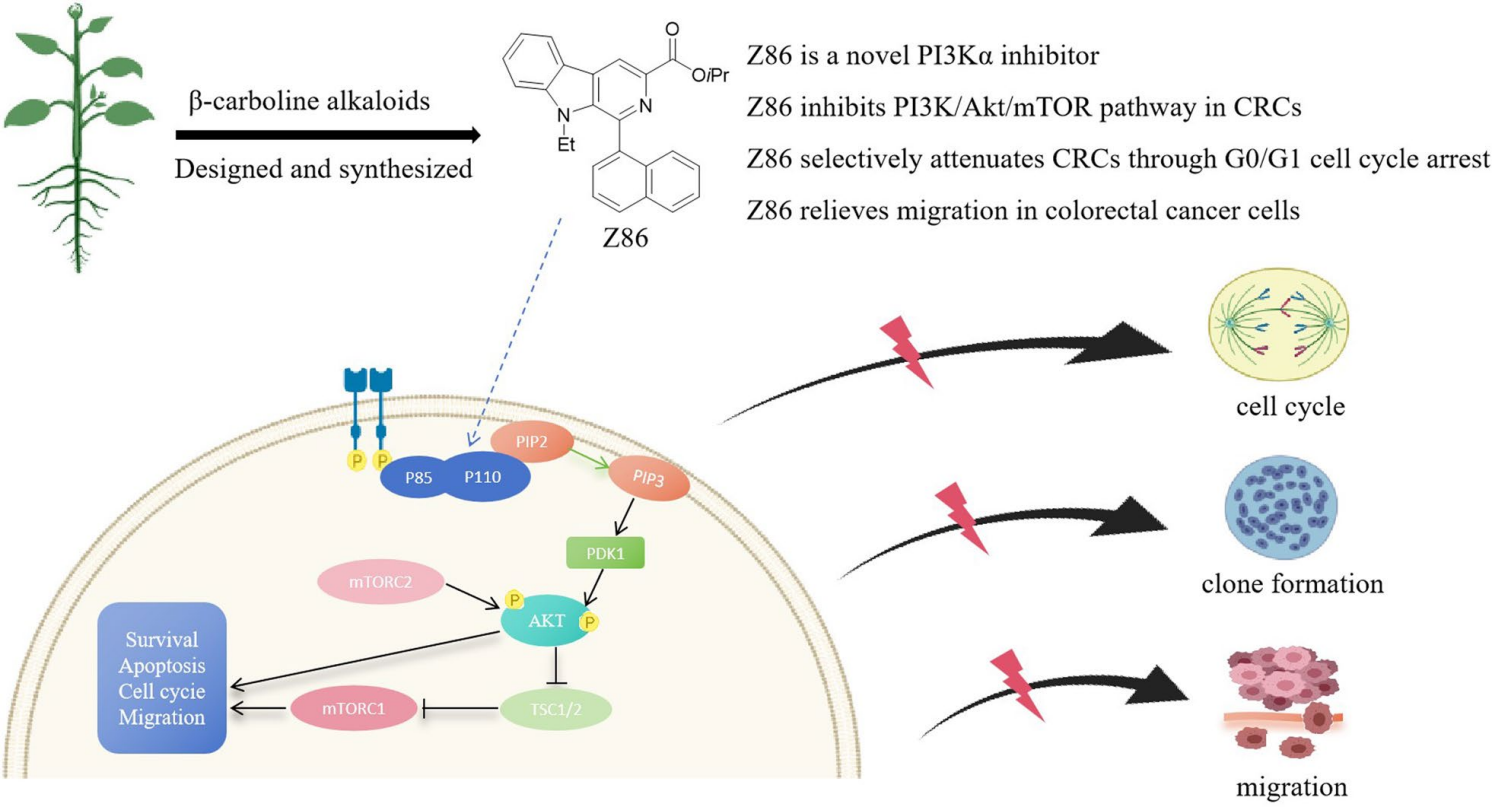

## Introduction

Colorectal cancer (CRC) ranks a leading common malignancy with high morbidity and mortality [1]. In recent years, with the development of chemotherapy drugs, the survival improvement for colorectal cancer patients has been achieved. However, the prognosis for advanced, recurrent and metastatic CRCs are not satisfactory, with current limited treatment options [2]. A number of genetic driven mutations for CRCs have been revealed with genomic analysis, among which PI3K/Akt/mTOR signaling is vital in colorectal development [3–5]. The stimulation of extracellular factors via receptor tyrosine kinases, PI3K amplification and mutation, as well as Ras induced activation of PI3K significantly promotes the proliferation of CRCs [6]. In addition, PI3K also contributes to acquired drug resistance of CRCs [7, 8]. More encouragingly, PI3K antagonists have been approved by FDA for clinical applications and a number of PI3K inhibitors are under clinical trials. Due to its key roles in initiation and progression, PI3K may serve as a striking therapeutic target for CRCs.

Several potent natural and synthetic molecules have been recognized to suppress CRCs by effectively blocking the PI3K pathway. The β-carboline alkaloids were firstly extracted from natural alkaloids and possess diverse pharmacological properties, including anticarcinogenic, anticardiovascular, antibacterial and antiviral activities [9–11]. In recent decades, a variety of novel β-carborinoids with antitumor activity have been designed and synthesized, while unique chemicals of β-carbolines, such as harmine, were found to overcome resistance to mitoxantrone and camptothecin [12]. Z68, isopropyl 9-ethyl-1-(naphthalen-1-yl)-9 H-pyrido[3,4-b]-indole-3-carboxylateis, was a β-carboline derivative we previously discovered with anti-CRCs activity and Wnt Signaling inhibitory activity [13], while its accurate target remains unclear. Here, we identified Z86 as a novel PI3Kα inhibitor. Our further study found Z86 disrupted the constitutive activation of PI3K/Akt/mTOR and relieved CRCs through G0/G1 cycle arrest and the migration as well.

## Results

### Z86 is a novel PI3Kα inhibitor

**Fig. 1:**
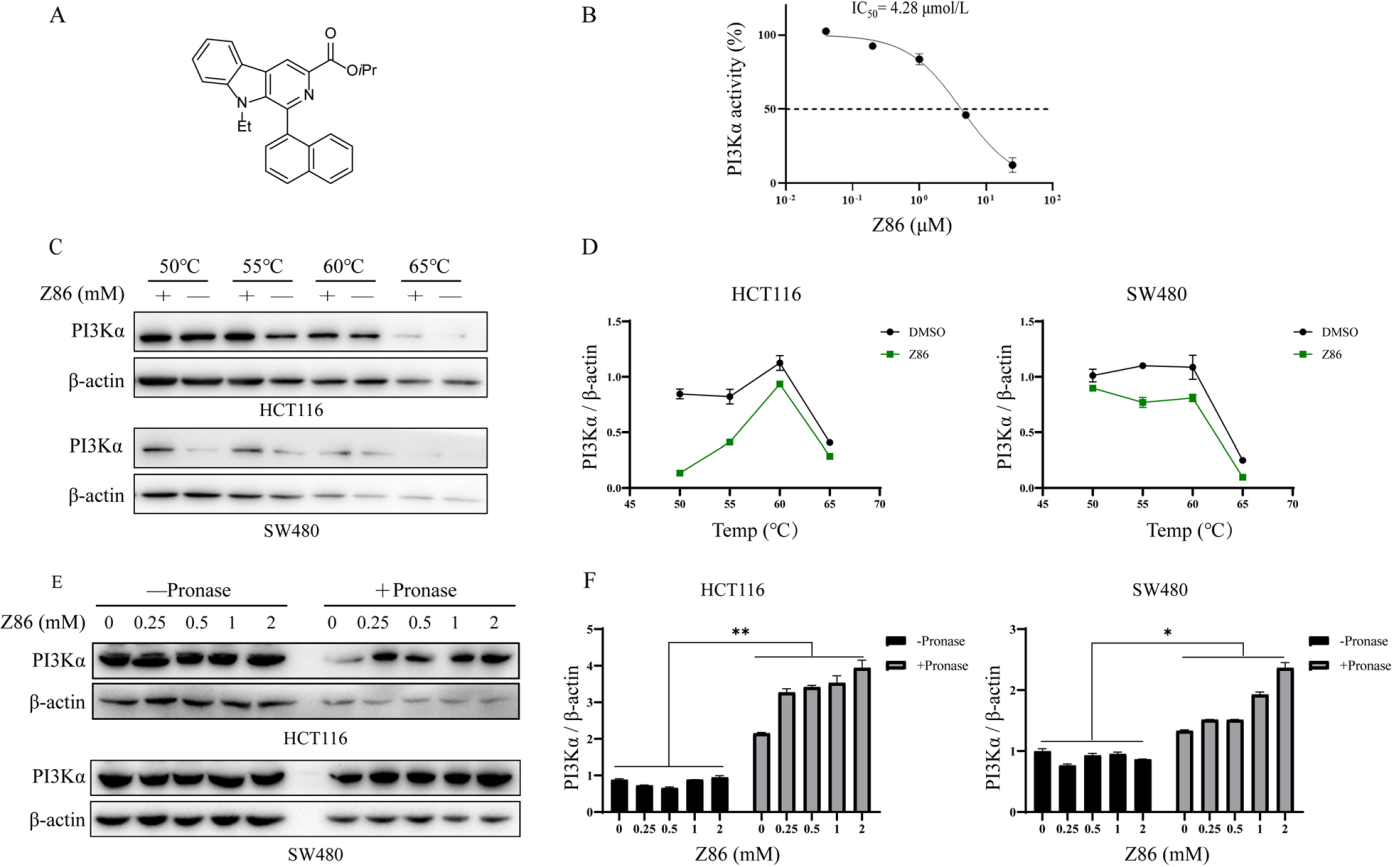
Z86 is a novel PI3Kα inhibitor. **A** The structure of compound Z86. **B** In vitro PI3Kα inhibitory of Z86. Purified PI3Kα was pretreated with Z86 and the activity of PI3Kα was evaluated. **C** CETSA was conducted with of HCT116 or SW480 cell lysates treated with Z86 with increasing melting temperatures (50-65 ℃, interval temperature: 5 ℃). **D** CETSA curves in cell lysates treated with Z86 or DMSO. **E** Immunoblot analysis of PI3Kα treated with DARTS assay in HCT116 or SW480 cells. **F** DARTS curve was performed using GraphPad Prism software

Class I PI3Ks differ in terms of tissue distribution and function, and PI3Kα is believed to be ubiquitously expressed and essential for tumorigenesis. Based on the PI3K-Kinase Activity ELISA kit, we found Z86 (Fig. 1A) could inhibit PI3Kα kinase activity (Fig. 1B), with an IC_50_ of 4.28 µM. Binding of chemicals to the target protein appears to improve the thermal stability and relative protease resistance [14, 15]. CETSA (cellular thermal shift assay, CETSA) is widely used to verify the thermal stabilization improvement under heating conditions on binding of small molecules to target proteins [16]. We performed CETSA to further confirm the interaction between Z86 with PI3Kα. Compared to the DMSO group, Z86 treatment in HCT116 and SW480 cell lysates increased the thermal stability of PI3Kα at different temperatures (50 ℃, 55 ℃, 60 ℃ and 65 ℃) (Fig. 1C, D). Furthermore, protease induced degradation of target protein can be prevented when the ligand binds with target protein. As shown in Fig. 1-E and *F*, pronase induced proteolytic of PI3Kα, while the presence of Z86 partially protected PI3Kα degradation by pronase in the DARTS (drug affinity responsive target stability, DARTS) assay, while negative control β-actin was not affected by Z86. Collectively, PI3Kα was the direct target of Z86 in CRCs.

### Z86 inhibits PI3K/Akt/mTOR pathway in CRCs

**Fig. 2:**
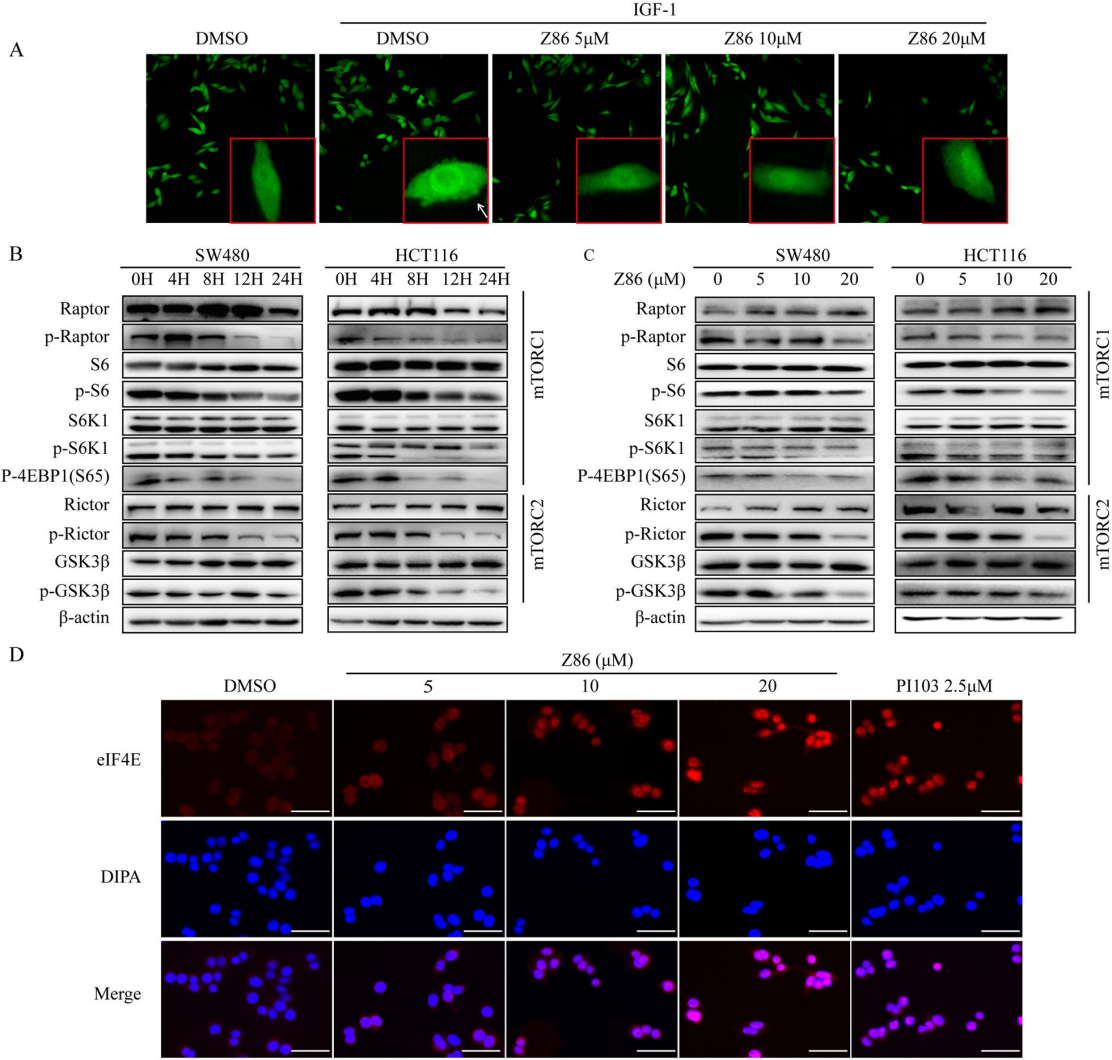
Z86 inhibits PI3K/Akt/mTOR pathway in CRCs. **A** The Akt1 redistribution change of Z86 on cells pretreated with IGF. Arrows indicate IGF-1-induced membrane aggregation. **B**, **C** The PI3K pathway downstream proteins in HCT116 and SW480 cells treated with 20 µM Z86 for indicated time or increasing concentrations. **D** Representative images of subcellular distribution of eIF4E in SW480 cells treated by Z86 for 8 h, and scale bar represents 50 μm

The PI3K/Akt/mTOR pathway is constitutively activated through various mechanisms. PI3K induced generation of phosphatidylinositol-3,4,5-trisphosphate recruits Akt to the inner plasma membrane and allows the activation of Akt, resulting in activation of a panel of downstream intracellular proteins [17–19]. Therefore, the AKT translocation in the plasma membrane is a marker of the of PI3K pathway activation, and the Akt distribution was detected in CHO cells with stable EGFP-Akt1 expression. As shown in Fig. 2A, IGF-1 significantly triggered plasma membrane translocation of Akt, however, Z86 significantly inhibited IGF-induced Akt aggregation in cell membrane, further confirming the PI3K inhibitory activity of Z86.

mTOR is a typical serine/threonine kinase can form multiprotein complex mTORC1 and mTORC2 with distinct structure and function. mTORC1 is the key activated downstream of PI3K, while several studies suggest that PIP3 promotes mTORC2 activation. To detect whether Z86 perform PI3K inhibitory activity through both the mTORC1 and mTORC2 components, western blot analysis was undertaken in CRCs. Strikingly, Z86 potently disrupted the phosphorylation of Raptor, S6K1, ribosomal S6 and 4E-BP1 in mTORC1 complex. Moreover, the phosphorylation of the key mTORC2 component Rictor and its downstream GSK3β at Ser9 was reduced in a concentration-dependent manner across all test units, which further indicated that Z86 effectively inhibited PI3K/Akt/mTOR signaling (Fig. 2B, C).

eIF4E protein is a key factor in translation regulation and is mainly distributed in the cytoplasm of mammalian cells. Once PI3K/Akt/mTOR signaling is inhibited, the eukaryotic initiation factor 4E (eIF4E)-binding protein 1 (4E-BP1) is failed to be phosphorylated by mTOR, and then eIF4E accumulates in the nucleus [20]. We examined whether Z86 affected the localization of eIF4E. As show in Fig. 2D, eIF4E in untreated HCT116 and SW480 cells was mainly distributed in cell cytoplasm. However, when treated with Z86, the eIF4E nuclear translocation increased significantly. Altogether, the results suggested Z86 inhibited PI3K/Akt/mTOR signaling activation in CRCs.

### Z86 selectively induces G0/G1 arrest to inhibit the proliferation of CRCs

**Fig. 3:**
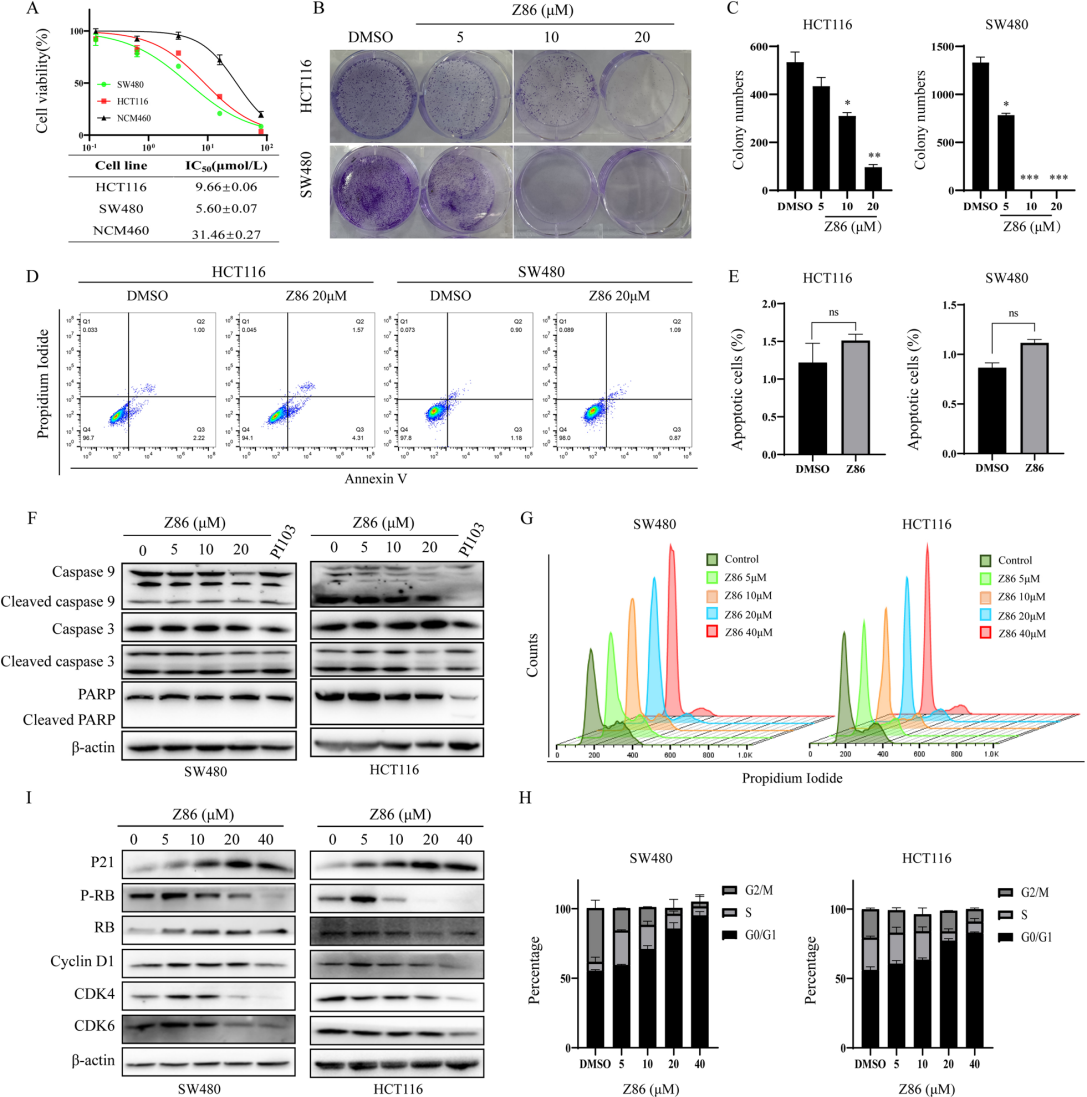
Z86 selectively induces G0/G1 arrest to inhibit the proliferation of CRCs. **A** The growth of HCT116, SW480 and NCM460 cells exposed to Z86 at 48 h was determined by MTS assay. **B**, **C** The colony formation inhibitory effect of Z86 was examined in CRCs, and the number of colonies was quantified. **D**, **E** The apoptosis analysis was performed in cells administrated with Z86 at 20 µM for 48 h. **F** The cleavage of PARP, Caspase 3 and Caspase 9 was detected in Z86 exposed CRCs. **G**, **H** Cell cycle distribution was undertaken by with propidium iodide staining in Z86 exposed CRCs. Cell cycle distribution was quantified. **I** Cell cycle-related proteins were blotted in cells incubated with Z86 for 24 h, and β-actin is the loading control

Since PI3K/Akt/mTOR signaling is vital in cell proliferation regulation, MTS and colony formation assays were performed to investigate whether Z86 inhibits cell proliferation of HCT116 and SW480 with dysregulated PI3K signaling pathway and human colon mucosal epithelial cells NCM460. As shown in Fig. 3A, Z86 exhibited concentration-dependent growth inhibition against HCT116 and SW480 cell lines, and the IC_50_ values at 48 h were 9.66 ± 0.06 µM and 5.60 ± 0.07 µM in the MTS assay, respectively. However, Z86 demonstrated little cytotoxicity towards NCM460 cells (IC_50_, 31.46 ± 0.27 µM), suggesting the hypo-toxicity of Z86 to normal cells. Furthermore, the colony formation ability was significantly inhibited in Z86 administrated HCT116 and SW480 cells, while the colony size was also much smaller compared with the control group (Fig. 3B, C). Taken together, Z86 selectively suppressed CRCs with constitutive PI3K/Akt/mTOR signaling pathway.

**Fig. 4:**
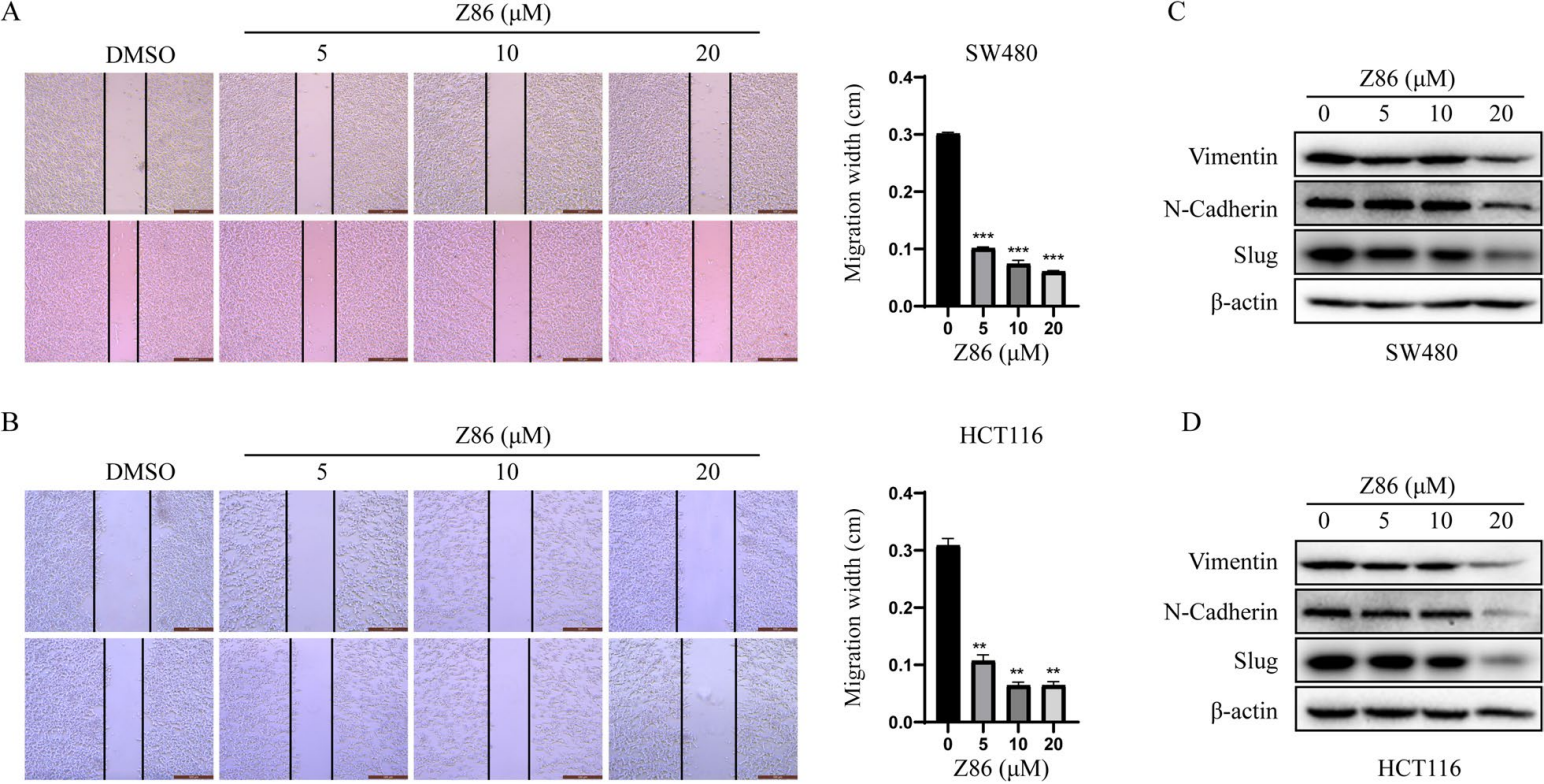
Z86 inhibits migration in CRCs. **A**, **B** SW480 and HCT116 cells were exposed to Z86, and the representative images were taken respectively. Scale indicates 500 μm. **C**, **D** The EMT-related proteins in cells treated with Z86 were investigated with western blot analysis

Generally, both apoptosis and cycle arrest lead to proliferation inhibition, the proliferation inhibitory mechanism of Z86 was investigated. Firstly, the involvement of apoptosis in Z86 induced cell proliferation regression in CRCs was examined. HCT116 and SW480 cells were administrated with 20 µM Z86 for 48 h, while hardly apoptotic cells were detected in the apoptosis analysis assay (Fig. 3D, E*)*. Furthermore, the apoptosis hallmarks of cleavage of apoptosis related proteins PARP, caspase 9 and caspase 3, were not induced in Z86 exposed CRCs (Fig. 3F). Thus, to further determine the mechanism of Z86 triggered CRCs suppression, we analyzed the distribution of cell phase with propidium iodide staining. The cell cycle analysis showed that Z86-treated HCT116 and SW480 cells exhibited marked increase of the G0/G1 cells, especially at concentrations of 20 and 40 µM (Fig. 4G, H). Altogether, Z86 induced proliferation inhibition of CRCs in a G0/G1 cell cycle arrest depending manner without inducing apoptosis. PI3K/Akt/mTOR signaling pathway regulates its downstream cell cycle related proteins of cyclin D1 and p21, followed by their heterodimerization with CDK4/6 to phosphorate Rb and subsequent G1/S transition [21, 22]. Therefore, the cell cycle-related proteins mentioned above was investigated by immunoblot analysis in CRCs treated with Z86. As shown in Fig. 4I, Z86 treatment up-regulated the CDK inhibitory protein p21 and down-regulated Cyclin D1, CDK4/6 and Rb phosphorylation. Collectively, the data above depicted that Z86 induced inactivation of PI3K/Akt/mTOR resulted in cyclin D1 reduction and p21 induction and subsequent dephosphorylation of p-RB, leading to G0/G1 arrest and proliferation inhibition.

### Z86 inhibits migration in Colorectal cancer cells

Accumulated evidence has suggested migration and invasion of CRCs is widely controlled by PI3K/Akt/mTOR signaling pathway [23]. As such, wound healing assay was conducted to test whether Z86 impaired the migration of CRCs. The migration distance at 24 h of both cell lines decreased with the increasing Z86 in Fig. 4A, B*.* Epithelial mesenchymal transition (EMT) is highly conserved in tumorigenesis and confers metastatic properties, which is promoted by PI3K/Akt activation [24]. PI3K/Akt/mTOR activation induced GSK-3β degradation and inhibition lead to the overexpression of Slug, which subsequently upregulates the mesenchymal cell markers, with N-cadherin and Vimentin included, thereby inducing EMT [25, 26]. Thus, the EMT biomarkers N-cadherin, vimentin and Slug were further examined. As shown in Fig. 4C, D, the proteins above were decreased in HCT116 and SW480 cells incubated with Z86 in western blotting assay. These data indicated that Z86 could suppress EMT and migration in CRCs.

## Discussion and conclusion

Colorectal cancer is one of the most common aggressive malignancies, and novel sensitive and targeted therapeutic agents are urgently needed. As the frequently dysregulated pathway in CRCs, PI3K is aberrantly activated through various mechanisms and vital to promoting the carcinogenesis and promotion of CRCs [27]. PIK3CA amplification and mutation occur in 30% CRCs. Ras, a small GTPase interacting and activating PI3K, is the frequently mutated genes in CRCs, with approximately 40% CRCs [28]. In this study, we found Z86, a β-carboline structure type compound, was identified as a novel PI3Kα inhibitor and selectively inhibited the proliferation of colorectal HCT116 (*PIKCA*^*H1047R*^, *KRAS*^*G13D*^) and SW480 (*PIK3CA* amplification, *KRAS*^*G12V*^) cells with aberrant PI3K activation. CETSA and DARTS demonstrated the enhanced thermal stability and resistance to pronase digestion, further confirming the interaction between Z86 and PI3Kα. Activation of PI3K recruits Akt to the membrane and its downstream protein phosphorylation, while Z86 inhibited membrane translocation of Akt and the downstream proteins of PI3K including mTORC1 related p-Raptor, p-S6, p-S6K1, p-4EBP1 and mTORC2 component p-Rictor, p-GSK3β, confirming that Z86 was a novel PI3Kα inhibitor. Besides, PI3K induced Akt activation leads to phosphorylation of GSK3β (Ser9) and GSK3β inhibition. And GSK3β is believed to phosphorylate β-catenin, resulting in β-catenin degradation and subsequent Wnt signaling repression [29]. We previously found Z86 induced the phosphorylation and subsequent degradation of β-catenin through inhibiting phosphorylation of GSK3β (Ser9) and inducing the hyperactivation of GSK3β [13]. The PI3Kα inhibitory activity provided a reasonable mechanism of Z86 on GSK3β and subsequent β-catenin degradation.

The PI3K/Akt/mTOR pathway tightly regulates cell proliferation, as well as apoptosis, and inhibition of PI3K is generally believed to attenuate tumor growth by apoptosis induction, since the important role of anti-apoptotic function of PI3K in tumorigenesis [30]. However, PI3K inhibitors such as ZSTK474S [31] and NVP-BEZ235 [32] have also been previously reported to exert their anti-tumor effects by blocking G0/G1 cell cycle arrest rather than inducing apoptosis, as PI3K regulates proliferation by promoting the G1/S phase transition [33]. In this study, we demonstrated that Z86 effectively induced G0/G1 arrest rather than apoptosis to inhibit the growth of CRCs. Further mechanism study showed Z86 increased the expression of CDK inhibitor p21 whereas the level of cyclin D1 and Rb phosphorylation in CRCs was reduced. These data suggested that Z86 induced PI3K inhibition and subsequent G0/G1 cell cycle arrest might contribute to tumor proliferation inhibition.

EMT is critical to the progression of cancer metastasis by converting epithelial cells into mesenchymal cells [34], and PI3K/Akt/mTOR signaling pathway regulates the EMT-associated genes through intracellular kinase cascades. Here, we demonstrate that Z86 treatment inhibits CRC migration with wound-healing assay, and the hallmarks of EMT, N-cadherin, Slug and Vimentin, were suppressed [35], indicating Z86 suppressed tumor migration.

In conclusion, our present study not only demonstrates that Z86 is a novel PI3Kα inhibitor with PI3K-mediated CRCs growth and migration inhibition, but also elucidates a reasonable molecular mechanism of Z86 in the Wnt signaling pathway inhibition.

## Experimental section

### Reagents

All the cell culture reagents were from HyClone (Logan, UT, USA). Antibodies against Raptor (#2280S), p-Raptor (#2083S), p70S6K1 (#2708S), p-p70S6K1 (#9206S), p-4E-BP1 (#13443S), Rictor (#2114), p-Rictor (#3806S), p-GSK3β (#9336L), p-S6 (#4858S), N-cadherin (#14215S), Slug (#9585S), caspase 3 (#14220S), p21 (#2947), CDK4 (#12,790), CDK6 (#13,331), p-Rb (#9308S) were from Cell Signaling Technology. Antibodies against GSK3β (22104-1-AP) and caspase 9 (10380-1-AP) were obtained from Proteintech. Antibodies specific for S6 (sc-74,459), vimentin (sc-32,322), PARP-1 (sc-8007), Rb (sc-74,562) and Cyclin D1 (sc-20,044) were procured from Santa cruz biotechnology. Antibody against β-actin (A1978) was supplied by Sigma. Z86 was obtained as previously reported [13].

### Cell culture

Colorectal SW480 cancer cells were produced by the American Type Culture Collection, while colon HCT116 cells and the normal human colon mucosal epithelial cells NCM460 were from Cell Bank of Chinese Academy of Sciences. All the cells were cultured according to the manufacture’ protocols.

### PI3K activity assay

The PI3Kα inhibitory activity of Z86 was performed as previously with the PI3-Kinase Activity ELISA kit (K-1000s, Echelon) based on the production of PIP3 [36].

### CETSA assay

HCT116 and SW480 cells treated with Z86 for 4 h were collected and exposed to heat shock at 50–65 °C for 3 min, followed by being lysed. After centrifuging, the related proteins were detected with western blot analysis.

### DARTS assay

SW480 and HCT116 cells were collected, then lysed with M-PER lysis solution on ice. After centrifugation, the supernatant was subjected to protein quantification. Cell lysate was incubated with Z86 at 4 ℃ for 12 h, followed by digestion with pronase (Roche). The digestion reaction samples stopped by the protease inhibitor cocktail were analyzed with Western blot analysis after 5 × loading buffer was added [37].

### Cytotoxicity assay

Attached cells were exposed to Z86 with different doses for indicated time, then 100 µl cultural medium containing 20 µl MTS solution (Promega) was added to each well and further cultured for 1 h at 37 °C. The absorbance value at 490 nm was determined. Three independent experiments were performed.

### Colony formation assay

Cells were exposed to Z86 for 24 h and were further grown without Z86 for another 13 days, then fixed and incubated with crystal violet solution. The colony numbers (> 50 cells) were documented.

### Wound Healing scratch assay

The migration of the cells incubated with Z86 for 24 h was analyzed and calculated with the Image J software based on the distance between the wound sides.

###  Western blot analysis

The whole protein of CRCs treated with Z86 was obtained using the RIPA buffer. After protein quantification, the protein was then separated on SDS-PAGE and transferred to PVDF membranes (Millipore). These membranes were immediately blocked and incubated overnight with specific antibodies, then incubated with the corresponding secondary antibody and subjected to chemiluminescence identification.

### Immunofluorescence, cell cycle and apoptosis analysis

The immunofluorescence detection of eIF4E, the cell cycle and apoptosis analysis of cells treated by Z86 were performed as previously [36]. The flow cytometry was performed by FACSCelesta (BD Biosciences). The anti-eIF4E antibody (#610,269, BD) and the Annexin V/FITC apoptosis Kit (4 A Biotech) were used according to the manufactures’ instructions.

### Data analysis

All data were presented as mean ± standard deviation (SD) of three separate experiments with statistical significance evaluated by Student’s *t*-test, and when the p value less than 0.05 was considered significant. Data derived graphs were obtained from GraphPad Prism 5.0 software.

## Data Availability

All the data supporting the findings of this study are available from the corresponding author on reasonable request.
